# Prognostic and clinical value of serum albumin, cortisol and TNF-a in treatment selection for advanced ovarian cancer patients

**DOI:** 10.5937/jomb0-57224

**Published:** 2025-11-05

**Authors:** Rong Jiang, Xin Pan, Xiu Shi, Jinhua Zhou, Juan Wang, Hong Zhang, Ma Jingjing

**Affiliations:** 1 The First Affiliated Hospital of Soochow University, Department of Gynaecology and Obstetrics, Suzhou, Jiangsu 215000, China

**Keywords:** serum cortisol, TNF-a, albumin, advanced ovarian cancer, serum albumin, R0, complications, overall survival, serumski kortizol, TNF-a, albumin, uznapredovali karcinom jajnika, serumski albumin, R0, komplikacije, ukupno preživljavanje

## Abstract

**Background:**

This study aimed to evaluate the prognostic significance of pre-treatment serum albumin levels and assess their association with clinical outcomes and surgical decisions in advanced ovarian cancer (O C) patients. Cortisol and tumour necrosis factor-alpha (TN F-a) were also measured to explore potential but secondary relationships.

**Methods:**

A retrospective analysis was conducted on OC patients undergoing surgery at our hospital from June 2022 to June 2024 with complete clinical and pathological data. Patients were categorised into low ALB (&lt; 35 g/L) and normal ALB (&gt;35 g/L) groups based on pre-treatment serum ALB levels. Each group was further divided into primary debulking surgery (PDS) and neoadjuvant chemotherapy, followed by interval debulking surgery (NACT-IDS) subgroups. Clinicopathologic characteristics were analysed.

**Results:**

Pre-treatment serum ALB levels positively correlated with progression-free survival (PFS) (r= 0 .2 9 8 9 , P&lt; 0.05) and overall survival (OS) (r= 0.2702, P&lt; 0.05), with the low ALB group exhibiting significantly lower PFS and OS (P&lt; 0.05). In the low ALB group, significant differences were observed between PDS and NACT-IDS in ascitic fluid level, R0 cytoreduction rate, postoperative complications, and length of stay (P&lt; 0.05), though PFS and OS were comparable (P&gt; 0.05). Similarly, in the normal ALB group, significant differences were noted in FIGO staging, ascitic fluid level, haemoglobin (HGB) levels, intra-operative haemorrhage, blood transfusion volume, R0 cytoreduction rate, and length of stay (P&lt; 0.05), while PFS and OS remained unaffected by treatment type (P&gt; 0.05). Additionally, C-reactive protein (CRP) levels were significantly higher in the low ALB group (6.5± 1.1 mg/L vs 5.2± 1.0 mg/L, P&lt; 0.05), indicating greater inflammation, whereas HGB levels were substantially lower (110.4± 15.8 g/L vs 118.7± 10.5 g/L, P= 0.021), reflecting poorer nutritional status. TNF-a levels showed a non-significant upward trend (P= 0.058), while cortisol levels were similar between groups (P= 0.073).

**Conclusions:**

Selecting NACT-IDS for advanced OC patients with hypoalbuminemia may help reduce the incidence of postoperative complications and improve the likelihood of achieving optimal cytoreduction. While survival outcomes (PFS and OS) did not significantly differ between treatment approaches in this study, the observed surgical benefits suggest that hypoalbuminemia could be considered a supportive factor in treatment planning. However, this indicator should be used cautiously and in conjunction with other clinical parameters until further evidence is available. The roles of TNF-a and cortisol in this context remained inconclusive and warrant further investigation.

## Abstract (sr)

**Uvod:**

Ova studija je imala za cilj da proceni prognostički značaj nivoa serumskog albumina pre započinjanja terapije i ispita povezanost sa kliničkim ishodima i hirurškim odlukama kod pacijentkinja sa uznapredovalim karcinomom jajnika (OC). Takođe su mereni kortizol i faktor nekroze tumora-alfa (TNF-a) kako bi se ispitali moguci sekundarni odnosi.

**Metode:**

Sprovedena je retrospektivna analiza pacijentkinja sa OC koje su operisane u našoj ustanovi od juna 2022. do juna 2024. godine, sa kompletnim kliničkim i patohistološkim podacima. Pacijentkinje su bile podeljene u grupe sa niskim ALB (&lt; 35 g/L) i normalnim ALB (&gt;35 g/L) nivoima na osnovu vrednosti serumskog ALB pre početka lečenja. Svaka grupa je dalje podeljena na podgrupe koje su lečene primarnom citoreduktivnom hirurgijom (PDS) i neoadjuvantnom hemioterapijom pracenom intervalnom citoreduktivnom hirurgijom (NACT-IDS). Analizirane su kliničko-patološke karakteristike.

**Rezultati:**

Nivoi serumskog ALB pre lečenja su pokazali pozitivnu korelaciju sa preživljavanjem bez progresije bolesti (PFS) (r= 0,2989, P&lt; 0,05) i ukupnim preživljavanjem (OS) (r= 0,2702, P&lt; 0,05), pri čemu je grupa sa niskim ALB imala značajno niže PFS i OS (P&lt; 0,05). U grupi sa niskim ALB su uočene značajne razlike između PDS i NACT-IDS u nivou ascitesa, stopi R0 citoredukcije, postoperativnim komplikacijama i dužini hospitalizacije (P&lt; 0,05), dok su PFS i OS bili slični (P&gt; 0,05). Slično tome, u grupi sa normalnim ALB su zabeležene značajne&nbsp;razlike u FIGO stadijumu, nivou ascitesa, vrednostima hemoglobina (HGB), intraoperativnom krvarenju, količini transfuzije, stopi R0 citoredukcije i dužini hospitalizacije (P&lt; 0,05), dok PFS i OS nisu zavisili od vrste terapije (P&gt; 0,05). Takođe, vrednosti C-reaktivnog proteina (CRP) su bile značajno vise u grupi sa niskim ALB (6,5± 1,1 mg/L naspram 5 ,2 ± 1 ,0 m g/L, P &lt; 0 ,0 5 ), sto ukazuje na izraženiju upalu, dok su vrednosti HGB bile znatno niže (1 10,4± 1 5,8 g/L naspram 11 8,7± 10,5 g/L, P= 0,021), sto odražava losiji nutritivni status. Vrednosti TNF-a su pokazale neznatan trend porasta (P = 0,058), dok su vrednosti kortizola bile slične između grupa (P= 0,073).

**Zaključak:**

Izbor NACT-IDS kod pacijentkinja sa uznapredovalim OC i hipoalbuminemijom može pomoci u smanjenju postoperativnih komplikacija i povecanju sanse za postizanje optimalne citoredukcije. Iako se preživljavanje (PFS i OS) nije značajno razlikovalo u zavisnosti od terapijskog pristupa u ovoj studiji, uočene hirurske koristi ukazuju da hipoalbuminemija može biti koristan dodatni faktor u planiranju lečenja. Ipak, ovaj indikator treba koristiti oprezno i u kombinaciji sa drugim kliničkim parametrima dok se ne dobiju dodatni dokazi. Uloge TNF-a i kortizola u ovom kontekstu ostaju nejasne i zahtevaju dalje istraživanje.

## Introduction

Ovarian cancer (OC) is one of the three common malignancies in gynaecology [1] [2]. Due to the fact that ovaries present a deep location in the pelvic cavity and specific early diagnostic methods are lacking, most OC patients are already in advanced stages when seeing a doctor, with a 5-year survival rate hovering at 30%-40% [3] [4]. The standard treatment plan for advanced OC is surgery + chemotherapy±maintenance therapy, such as primary debulking surgery (PDS) supplemented with platinum-based post-operative chemotherapy [5] [6] [7]. To achieve an ideal cytoreductive effect, it is necessary to resect as many resectable metastatic lesions and organs as possible, including invaded small and large intestinal segments, as well as liver, spleen, etc. This not only requires surgeons to have superb skills and extraordinary patience but also requires patients' physical condition to be able to withstand surgery. For patients with high perioperative risk or difficulty in achieving satisfactory cytoreductive effect (R0: no visible residual lesions after surgery, or R1: residual lesions less than 1 cm after surgery), neoadjuvant chemotherapy followed by interval debulking surgery (NACT-IDS) supplemented with platinum-based postoperative chemotherapy should receive selection [8] [9]. Neoadjuvant chemotherapy can effectively elevate the tumour resection rate of advanced OC patients and markedly attenuate the risk of surgical complications and perioperative mortality. However, it does not improve survival; it is still another option for advanced OC patients who cannot undergo PDS smoothly [10]. Nevertheless, screening suitable treatment options for advanced OC patients remains an urgent problem that needs to be solved.

Serum albumin (ALB) level is an effective marker for judging malnutrition in patients with malignancies and can, to some extent, determine the nutritional status of patients [11] [12], while OC is the gynaecological malignant tumour most closely related to hypoalbuminemia [13] [14]. Reports have demonstrated that serum ALB level is an independent prognostic element for OC [15] [16]. Thus, serum ALB is of great significance for the pre-treatment evaluation of patients with advanced OC.

This research aimed to elucidate the evaluation value of serum ALB in selecting treatment methods for advanced OC patients, which may provide a reference for surgical procedures for advanced OC patients.

## Materials and methods

### Study design and patient selection

This retrospective study included ovarian cancer (OC) patients who underwent surgery at the First Affiliated Hospital of Soochow University between June 2022 and June 2024. Eligible patients were required to have complete clinical and pathological data.

The inclusion criteria were:

1. Pathological diagnosis of stage III-IV epithelial ovarian cancer based on FIGO classification, confirmed either at our hospital or another tertiary medical centre;
2. Underwent either primary debulking surgery (PDS) or neoadjuvant chemotherapy followed by interval debulking surgery (NACT-IDS), which involved procedures such as panhysterectomy, bilateral adnexectomy, pelvic and para-aortic lymphadenectomy, greater omentum resection, appendectomy, and resection of abdominopelvic implant lesions;
3. Availability of a pre-treatment serum albumin (ALB) level measured within one week prior to the initiation of treatment.

The exclusion criteria were:

1. Coexisting severe infection, haematological disorders, autoimmune diseases, or other malignancies;
2. Presence of liver cirrhosis, hepatitis, liver metastases, or other severe hepatic conditions.

Our hospital's Ethics Committee approved the study.

### Data collection and grouping

Demographic and clinical data were collected for each patient, including age, height, weight, FIGO stage, histopathologic subtype, tumour grade, pretreatment haemoglobin (HGB), cytoreductive outcome, serum ALB levels, operative time, intraoperative blood loss, transfusion volume, postoperative complications, and length of hospital stay.

Patients were stratified into two groups based on their serum ALB levels prior to treatment:

- Low ALB group: ALB<35 g/L
- Normal ALB group: ALB≥35 g/L

Each group was further divided into subgroups based on the surgical approach received (PDS or NACT-IDS). For patients undergoing NACT-IDS, hypoalbuminemia was corrected before surgery [17].

### Follow-up

Patients were followed until June 2023. Follow-up was conducted every 3 months for the first two years after treatment, every 6 months for the next three years, and annually thereafter. Follow-up methods included outpatient visits and telephone interviews, with some assessments performed at local hospitals. Routine follow-up evaluations included gynecologic examination, abdominal ultrasound or CT, chest imaging, and tumour marker analysis. MRI was conducted when clinically necessary.

### Statistical analysis

Statistical analyses were performed using SPSS version 27.0. Survival time was measured from the date of pathological diagnosis to either the date of death or last follow-up for overall survival (OS) and to the date of recurrence for progression-free survival (PFS).

Quantitative data were expressed as mean±standard deviation (SD). For group comparisons, independent sample t-tests were used for normally distributed data, and Mann-Whitney U tests were used for non-normally distributed data. Categorical variables were expressed as numbers and percentages [n (%)] and compared using the chi-square test.

Quantitative data were expressed as mean± standard deviation (SD). For group comparisons, independent sample t-tests were used for normally distributed data, and Mann-Whitney U tests were used for non-normally distributed data. Categorical variables were expressed as numbers and percentages [n (%)] and compared using the chi-square test.

## Results

### Clinicopathologic characteristics of patients

A total of 214 patients were included in this research, of which 14 cases were lost to follow-up (6.54%), and 200 cases were followed up (93.46%). Among 200 cases, mean age was (52.31 ±7.07) years old; body mass index (BMI) was (23.06±2.49) kg/m^2^; The serum ALB value before the first treatment was (37.77±6.14) g/L; length of stay was (15.56±3.05) days (Table 1).

**Table 1:** Clinicopathologic characteristics of 200 cases.

Characteristics	N	Age (years)	BMI (kg/m^2^)	Serum ALB level before<br>treatment (g/L)	Length of stay (d)
	200	52.31±7.07	23.06±2.49	37.77±6.14	15.56±3.05

### Analysis of the correlation of serum ALB and various clinical characteristics

**Figure 1:**
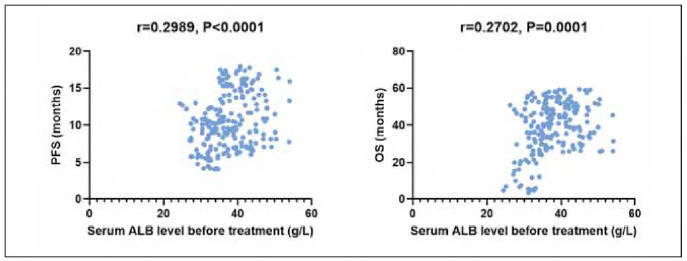
Correlation of serum ALB and various clinical characteristics.

Correlation analysis results demonstrated a positive correlation between pre-treatment serum ALB level and PFS (r=0.2989, P<0.05) and a positive correlation between pre-treatment serum ALB level and OS (r=0.2702, P<0.05; Figure 1).

### Comparison of general data of patients with different serum ALB levels

**Figure 2:**
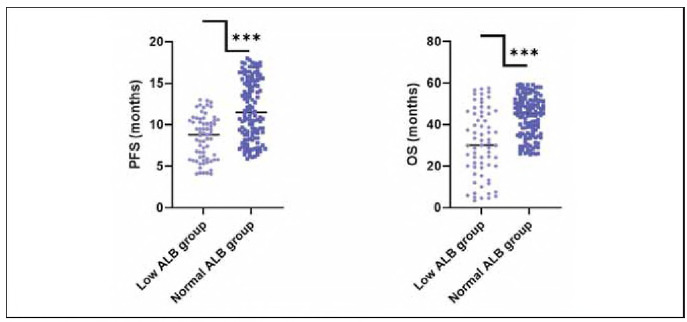
General data of patients with different serum ALB levels

According to pre-treatment serum ALB level, there were 67 cases (33.50%) in the low ALB group and 133 cases (66.50%) in the normal ALB group. The PFS and OS in the low ALB group were significantly lower than those in the normal ALB group, indicating statistical significance (P<0.05; Figure 2).

### Comparison of clinical data of patients undergoing two different surgical methods

There exhibited statistical significance between patients receiving PDS and those receiving NACT-IDS in low ALB group in terms of ascitic fluid level, proportion of cytoreductive effect reaching R0, incidence of postoperative complications, and length of stay (P<0.05), while no statistical significance exhibited in PFS and OS between patients receiving PDS and those receiving NACT-IDS in low ALB group (P>0.05). There exhibited statistical significance between patients receiving PDS and those receiving NACT-IDS in the normal ALB group in terms of FIGO staging, ascitic fluid level, pre-treatment HGB level, intraoperative haemorrhage volume, intraoperative blood transfusion volume, proportion of cytoreductive effect reaching R0, and length of stay (P<0.05), while no statistical significance exhibited in PFS and OS between patients receiving PDS and those receiving NACT-IDS in normal ALB group (P>0.05; Table 2).

**Table 2:** Clinical data of patients undergoing two different surgical methods.

Characteristics		Low ALB group		χ^2^/t	P	Normal ALB group		χ^2^/t	P
		PDS (n=21)	NACT-IDS<br>(n=46)			PDS (n=55)	NACT-IDS<br>(n = 78)		
Age (years)		50.10±8.84	51.61±6.78	0.471	0.639	53.60±7.05	54.12±6.86	0.046	0.964
BMI (kg/m^2^)		22.30±2.74	22.75±1.95	1.271	0.208	23.36±2.39	23.15±2.51	0.394	0.694
FIGO staging<br>[n (%)]	III C	19 (90.48)	36 (78.26)	1.463	0.226	52 (94.55)	58 (74.36)	9.19	0.002
	IV	2 (9.52)	10 (21.74)			3 (5.45)	20 (25.64)		
Histopathologic<br>types [n (%)]	Serous<br>carcinoma	16 (76.19)	39 (84.78)	0.724	0.395	46 (83.64)	60 (76.92)	0.898	0.343
	Non-serous<br>carcinoma	5 (23.81)	7 (15.22)			9 (13.36)	18 (23.08)		
Tumour<br>grading<br>[n (%)]	G1	2 (9.52)	2 (4.35)	1.61	0.447	3 (5.45)	3 (3.85)	0.898	0.826
	G2	0 (0.00)	0 (0.00)			0 (0.00)	1 (1.28)		
	G3	18 (85.72)	38 (82.61)			48 (87.28)	68 (87.18)		
	Unknown	1 (4.76)	6 (13.04)			4 (7.27)	6 (7.69)		
Ascitic fluid<br>level<br>[n (%)]	<2500 mL	10 (47.62)	43 (93.48)	18.344	<0.001	38 (69.09)	77 (98.72)	24.194	<0.001
	≥2500 mL	11 (52.38)	3 (6.52)			17 (30.91)	1 (1.28)		
HGB level before<br>treatment		118.00±10.20	115.90±6.80	0.052	0.959	122.36±9.18	116.30±6.77	4.006	<0.001
Operative duration<br>(min)		216.48±18.80	210.06±17.38	0.653	0.516	209.10±17.90	204.25±17.27	1.385	0.168
Intraoperative haemorrhage<br>volume (mL)		500.00±27.87	491.26±26.80	0.686	0.495	587.05±47.22	386.74±20.03	40.462	<0.001
Intraoperative blood<br>transfusion volume (mL)		461.00±39.04	453.15±32.31	1.584	0.118	418.26±40.11	175.12±18.21	41.685	<0.001
Resection rate<br>[n (%)]	R0	6 (28.57)	30 (65.22)	7.788	0.005	24 (43.64)	50 (64.10)	5.474	0.019
	R1+R2	15 (71.43)	16 (34.78)			31 (56.36)	28 (35.90)		
Postoperative<br>complications<br>[n (%)]	Presence	6 (28.57)	3 (6.52)	6.028	0.014	8 (14.55)	12 (15.38)	0.018	0.894
	Absence	15 (71.43)	13.20±1.89			47 (85.45)	66 (84.62)		
Length of stay (d)		18.40±2.55	13.20±1.89	9.539	<0.001	17.36±2.77	15.05±1.57	7.796	<0.001
PFS (months)		8.90±2.67	8.23±2.60	0.994	0.32	11.96±3.53	11.76±3.71	0.395	0.693
OS (months)		26.94±17.00	32.75±15.59	1.379	0.168	44.59±9.49	43.28±10.08	0.676	0.499

### Comparison of serum markers between study groups

In this study evaluating the role of ALB in selecting treatment methods for advanced ovarian cancer patients, the results highlighted significant differences between patients with low and normal ALB levels. C-reactive protein (CRP) levels were significantly higher in the Low ALB Group (6.5±1.1 mg/L) compared to the Normal ALB Group (5.2 ±1.0 mg/L, P<0.05), suggesting greater inflammation in hypoalbuminemia patients. TNF-α showed a non-significant trend towards higher levels in the Low ALB Group (22.3±4.5 ng/mL) compared to the Normal ALB Group (20.0±3.9 ng/mL, P=0.058). Similarly, Hemoglobin (HGB) levels were significantly lower in the Low ALB Group (110.4±15.8 g/L) compared to the Normal ALB Group (118.7±10.5 g/L, P=0.021), reflecting poorer nutritional status. Finally, Cortisol levels showed no significant difference between the groups (P=0.073), suggesting similar stress levels across both groups. These findings underscore the value of serum ALB as a prognostic marker in advanced OC, particularly for selecting treatment options to improve patient outcomes (Table 3).

**Table 3:** Comparison of serum markers between low ALB and normal ALB groups.

Serum Marker	Low ALB Group (Mean±SD)	Normal ALB Group (Mean±SD)	P-Value
CRP (mg/L)	6.5±1.1	5.2±1.0	<0.05
TNF-α (ng/mL)	22.3±4.5	20.0±3.9	0.058
HGB (g/L)	110.4±15.8	118.7±10.5	0.021

## Discussion

OC is a common gynaecological malignancy with the highest mortality rate; though the combination of multiple treatment modes has brought hope to patients with advanced OC, OS remains quite low [18]. Most advanced OC patients have a high tumour load and extensive metastasis in abdominopelvic cavities, and PDS is difficult to achieve a satisfactory cytoreductive level and is also prone to intraoperative bleeding and a high incidence of postoperative complications. In recent years, NACT-IDS has become one of the hot topics for researchers, and indications for NACT-IDS are constantly being updated and improved. Scholars, both domestically and internationally, have proposed that hypoalbuminemia should be one of the preferred indications for NACT-IDS in advanced OC patients [19], whereas ALB has not been validated as an independent element. This research evaluated the application value of serum ALB in different treatment options for advanced OC.

Serum ALB is the most abundant protein in human plasma synthesised by the liver; on the one hand, it exerts a crucial role in maintaining colloid osmotic pressure and can serve as a transport medium for intrinsic metabolites, drugs, and antioxidants; on the other hand, serum ALB can scavenge free radicals, repress platelet aggregation and anticoagulant physiological functions [20]. Thus, abnormal serum ALB levels can lead to imbalanced colloid osmotic pressure, resulting in ascites and an elevated risk of haemorrhage due to its inhibition of platelet aggregation and anticoagulant function. The diagnostic criteria for hypoalbuminemia in clinical practice are serum ALB levels below 35 g/L [17] [21]. Low serum ALB is also a reflection of inflammation and a risk element for postoperative complications and adverse prognosis of multiple cancers [22]. Moreover, low serum ALB is a recognised marker of malnutrition [23], and it is also a marker of systemic inflammatory activity. Thus, it can be applied as a predictor of postoperative complications and long-term survival of patients. Research has depicted that low serum ALB is regarded as a marker for screening surgical complications and higher perioperative mortality in inflammatory markers determining preoperative nutrition and predicting the perioperative prognosis of OC patients [24].

Herein, serum ALB level exhibited a positive correlation with patients' PFS and OS; that is, the higher the serum ALB, the longer the patients' PFS and OS, and PFS and OS in low ALB group exhibited depletion relative to those in the normal ALB group, further indicating that low serum ALB may be one of the elements for unfavourable prognosis of OC. In the low ALB group, with consistent baseline data such as age, FIGO staging, pathological types, and tumour grading, patients exhibited a higher R0 rate in NACT-IDS group relative to the PDS group; the incidence of postoperative complications in NACT-IDS group exhibited depletion relative to that in PDS group; cases with ascitic fluid volume ≥2500 mL and length of stay in NACT-IDS group exhibited depletion relative to those in PDS group, while no statistical significance in operative duration, intraoperative haemorrhage volume, intraoperative blood transfusion volume, PFS, and OS exhibited between both groups. These results may be related to the fact that advanced OC patients undergoing NACT-IDS have already received tumour-related and symptomatic supportive treatment before surgery; along with neoadjuvant chemotherapy, correction of ALB level improves patients' short-term nutritional status, making them more tolerant to surgery, and chemotherapy also attenuates size of lesions, thereby reducing ascites, surgical risk, and occurrence of postoperative complications. Furthermore, in the normal ALB group, the incidence of postoperative complications in patients receiving NACT-IDS was similar to those receiving PDS. This indicates that NACT-IDS does not elevate the incidence of postoperative complications in patients with normal ALB while improving the R0 rate, whereas it effectively attenuates the incidence of postoperative complications in patients with hypoalbuminemia. Thus, advanced OC patients with ALB levels below 35 g/L should first choose NACT-IDS to achieve a better cytoreductive effect and attenuate the incidence of postoperative complications. Moreover, in the normal ALB group, a proportion of stage IV patients in the NACT-IDS group exhibited elevation relative to that in the PDS group, while those with ascitic fluid volume ≥2500 mL had less intraoperative haemorrhage volume and blood transfusion volume, shorter length of stay, and higher R0 rate; nevertheless, no statistical significance in PFS and OS exhibited between NACT-IDS group and PDS group, which to some extent reflects advantages of NACT-IDS.

The observed correlation between serum albumin levels and survival outcomes in OC patients likely reflects a combination of nutritional and inflammatory mechanisms. Albumin is a well-established marker of nutritional status. Low ALB levels may indicate protein-energy malnutrition, which can impair immune response, wound healing, and tolerance to chemotherapy or surgery, thereby worsening prognosis [25]. However, hypoalbuminemia is also recognised as a negative acute-phase reactant, meaning its levels decline in response to systemic inflammation. In this study, the low ALB group exhibited significantly elevated CRP levels, supporting the notion that inflammation may contribute to reduced albumin synthesis or increased catabolism. Chronic inflammation in the tumour microenvironment is known to promote cancer progression, angiogenesis, and resistance to treatment [26]. Therefore, serum ALB likely reflects both the nutritional reserve and the degree of systemic inflammatory burden in OC patients, which together influence postoperative recovery and long-term survival.

Our findings align with previous studies that have established serum albumin as a significant prognostic marker in various malignancies. For instance, an earlier study reported that declining ALB levels during chemotherapy were associated with worse outcomes in cancer patients [27]. This supports our observation of shorter PFS and OS in the hypoalbuminemia group. Similar associations have also been documented in gastrointestinal, lung, and hepatocellular cancers, where low albumin predicted higher postoperative complication rates and reduced survival [28] [29]. These studies further underscore the dual role of ALB as both a nutritional and inflammatory indicator.

In contrast, TNF-α and cortisol levels did not differ significantly between groups in our study. This may be due to the complex and often transient nature of these biomarkers. TNF-α is known to have variable expression influenced by tumour type, disease stage, and host immune responses. While some studies have suggested a role for TNF-α in cancer-related cachexia and inflammation [30], its clinical utility as a prognostic marker in OC remains inconclusive. Similarly, cortisol levels can fluctuate due to stress, circadian rhythm, and medication use, potentially obscuring meaningful differences [31]. Thus, their non-significance in this cohort may reflect both biological variability and limited sensitivity in the context of advanced OC.

The postoperative complications of OC are mostly vaginal stump infection, intestinal obstruction, incision-related complications, and venous thrombosis; the occurrence of these complications has relation to multiple elements, among which patients' own nutritional status is a significant reason, and their own status has a close relation to ALB level [32]. This may be the reason for the high incidence of postoperative complications and slow recovery leading to extended hospital stays in advanced OC patients with low ALB. Nevertheless, a specific mechanism remains to be studied. It has been depicted that patients with postoperative cytoreductive effects reaching R0 in OC have more prolonged survival and greater benefits [33]. Though NACT-IDS did not exert a marked survival advantage over PDS in the low ALB group in this research, the surgical advantage was apparent, further confirming that NACT-IDS should be the first choice for patients with hypoalbuminemia. Nevertheless, the impact of serum ALB on the survival of patients with advanced OC was not validated in this research. Long-term follow-up should be conducted, and further large-scale, multicenter prospective studies should be carried out to confirm our findings.

However, it is essential to note the lack of statistically significant differences in PFS and OS between PDS and NACT-IDS subgroups in both the low and normal ALB groups. While this may appear to suggest equivalent long-term outcomes between these two surgical approaches, several contextual factors must be considered. First, the relatively short follow-up duration might not have been sufficient to capture survival differences, particularly in slowly progressing tumours. Second, patient selection bias may have affected these findings; for example, more patients with FIGO stage IV disease were observed in the NACT-IDS group within the normal ALB cohort. This may have diluted the potential survival benefit of NACT-IDS. Furthermore, while cortisol and TNF-α were examined, their levels showed no significant differences between groups, possibly due to sample size limitations or biological variability. Nonetheless, the non-significant trend toward elevated TNF-α in hypoalbuminemia patients may warrant further investigation, as it suggests an inflammatory component that could contribute to disease progression.

Another possible reason for the absence of significant survival differences is the inherent complexity of OC biology and its response to therapy. Cytoreductive success and complication rates are critical short-term metrics, but genetic heterogeneity, treatment adherence, recurrence patterns, and other comorbidities influence long-term outcomes. The observed benefits of NACT-IDS in terms of reduced ascites, blood loss, and hospital stay should not be underestimated, as these can substantially impact quality of life and recovery trajectory.

This study has several limitations that should be acknowledged. First, its retrospective design and single-centre setting may introduce selection bias and limit the generalizability of the findings. Second, the sample size, while adequate for preliminary analysis, remains relatively small for drawing definitive conclusions, mainly when subdivided into treatment groups. Third, although serum albumin was a central focus, other nutritional and inflammatory markers that may interact with ALB, such as prealbumin or lymphocyte counts, were not included. Furthermore, follow-up duration was limited to a short-term period, preventing robust evaluation of long-term survival outcomes. Future large-scale, multicenter prospective studies with longer follow-up and broader biomarker evaluation are needed to validate these findings and enhance clinical applicability.

## Conclusion

In conclusion, for advanced OC patients with hypoalbuminemia, NACT-IDS was associated with fewer postoperative complications and improved surgical outcomes, including a higher rate of complete cytoreduction. Although no significant differences in PFS or OS were observed between NACT-IDS and PDS in this study, the perioperative advantages of NACT-IDS suggest it may be a more suitable option for patients with compromised nutritional status. Therefore, serum albumin level may serve as a helpful reference indicator when tailoring surgical strategies, but further prospective studies are needed to confirm its role in guiding treatment decisions.

## Dodatak

### Acknowledgements

The authors would like to thank the staff of the Department of Gynaecology and Obstetrics, the First Affiliated Hospital of Soochow University, for their support in patient data collection and follow-up coordination.

### Funding

This work was supported by the Mechanism of targeting HK2 to induce DNA homologous recombination repair deficiency and enhance the sensitivity of ovarian cancer to PARP inhibitors (No. 82202898).

### Authors' contribution

Rong Jiang and Xin Pan contributed equally to this work and should be considered co-first authors. Jingjing Ma designed the study and supervised the research. Xiu Shi, Jinhua Zhou, and Juan Wang were responsible for data collection and patient follow-up. Hong Zhang performed the statistical analysis. Rong Jiang and Xin Pan drafted the manuscript. All authors read and approved the final manuscript.

### Ethical approval

The Ethics Committee of the First Affiliated Hospital of Soochow University approved this study. All procedures followed were in accordance with institutional guidelines and the Declaration of Helsinki.

### Informed consent

Informed consent was obtained from all individual participants included in the study.

### Data availability statement

The data supporting the findings of this study are available from the corresponding author upon reasonable request.

### Conflict of interest statement

All the authors declare that they have no conflict of interest in this work.

### List of abbreviations

OC, ovarian cancer;<br>ALB, albumin;<br>PDS, primary debulking surgery;<br>NACT-IDS, neoadjuvant chemotherapy followed by interval debulking surgery;<br>PFS, progression-free survival;<br>OS, overall survival;<br>FIGO, International Federation of Gynecology and Obstetrics;<br>HGB, haemoglobin;<br>CRP C-reactive protein;<br>TNF-α, tumour necrosis factor-alpha;<br>BMI, body mass index;<br>R0, no visible residual lesions after surgery;<br>R1, residual lesions less than 1 cm after surgery;<br>SPSS, Statistical Package for the Social Sciences.
